# Ferrofluid Droplet Chains in Thermotropic Nematic Liquid Crystals

**DOI:** 10.1002/cphc.202400858

**Published:** 2024-11-20

**Authors:** Varun Chandrasekar, Jian Ren Lu, Ingo Dierking

**Affiliations:** ^1^ Department of Physics and Astronomy University of Manchester Oxford Road Manchester M139PL UK

**Keywords:** Nematic liquid crystals, Ferrofluid, Chaining, Stokes velocity, Dipolar defects, Velocity of chain, Boundary layer, Surfactant

## Abstract

Dispersing ferrofluids in liquid crystals (LCs) produces unique systems which possess magnetic functionality and novel phenomena such as droplet chaining. This work reports the formation of ferrofluid droplet chains facilitated by the topological defects within the LC director field, induced by the dispersed ferrofluid. The translational and rotational motion of these chains could be controlled via application of external magnetic fields. The process of the droplet chain formation in LCs can be stabilized by the addition of surfactants. The magnetic colloidal particles in the ferrofluid located at the interface between the ferrofluid and the LC are arranged so that a boundary layer was formed. The velocities and boundary layer thickness values of ferrofluid droplet chains in nematic 5CB (4‐Cyano‐4′‐pentylbiphenyl) were investigated for varying average droplet sizes and number of droplets in a chain. The creation and behaviour of ferrofluid droplet chains in 5CB with the addition of the surfactant polysorbate 60 (Tween‐60) and without, was comparatively investigated. The integration of liquid crystals and ferrofluids along with the incorporation of functional materials facilitates the innovative development of advanced materials for future applications.

## Graphical abstract

Chaining of ferrofluid droplets via topological defects in a nematic liquid crystal and the corresponding dynamic behaviour of uncoated and amphiphilic coated chains under magnetic field application was investigated. The novel insights gained may open a pathway for new liquid crystal applications in the fields of sensing, micromanipulation and micro‐cargo delivery.

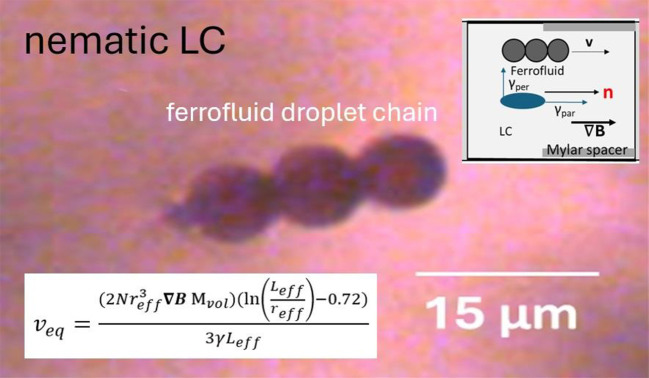

## 1 Introduction

Systematic exploration and investigation of a multitude of liquid crystal (LC)‐based materials incorporating dispersed nano‐ and micro‐particles constitutes a distinct modern field of LC science.[^1^, ^2^] The orientational ordering and relative translational freedom in LCs, combined with the properties of the dispersed materials, permits composites to have unique properties, which are not inherent to their components.^[3]^ LC‐composite materials have found applications in various fields such as nanophotonics,^[3]^ plasmonics,[^3^, ^4^] opto‐electronics,[^1^, ^3^, ^4^] photovoltaics,[^3^, ^4^, ^5^] metamaterials[^1^, ^4^] and sensors.^[3]^ LC emulsions can also be designed to respond to biomolecules, bacteria, or environmental pollutants, which make them suitable candidates for bio‐sensing applications.^[6]^

LC composites have given rise to considerable attention in areas that are especially related to non‐display technologies and nanotechnology. Doping of ferroelectric particles at low concentrations to a nematic LC may induce ferroelectric properties inherent to the particles.[^3^, ^8^] Low‐concentration LC nano‐colloids have potential for developing unique self‐organised materials and offer innovative and effective means to control precisely the physical properties of LCs.^[3]^ Mertelj et al.[^9^, ^10^] conducted experimental demonstrations confirming the theoretical prediction of a ferromagnetic nematic colloidal suspension. They demonstrated that an LC composite system doped with magnetic nanoparticles of specific geometry displays typical ferromagnetic properties, demonstrating hysteresis, magnetization reversal under an inverted external field, and showcasing domain walls and domain wall motions. The nematic composite material switches its magnetization at very small magnetic fields and may lead to new magneto‐optic devices.^[9]^ This ferromagnetic ordering is facilitated by the shape of the magnetic nanoparticles that have the form of thin platelets in contrast to what was theoretically predicted by Brochard and de Gennes where rod‐like magnetic particles dispersed in nematic and cholesteric LCs should lead to ferromagnetic systems.[^10^, ^11^]

**Figure 1:**
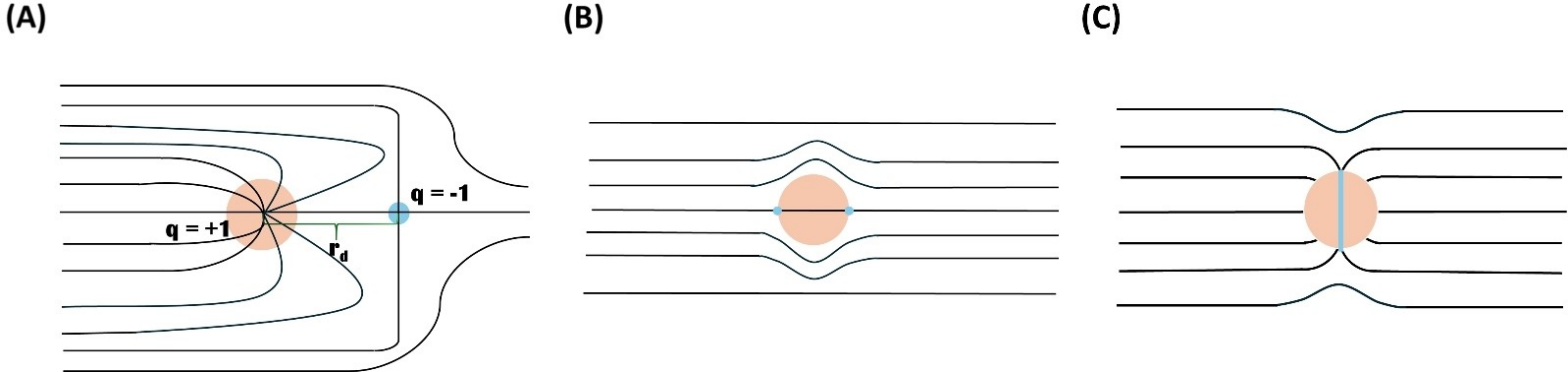
Schematic diagrams of defect formation in LCs due to the presence of colloidal particles (A) Formation of a hyperbolic hedgehog defect when a microsphere with homeotropic surface anchoring is inserted into a nematic liquid crystal (reproduced with permission from ref.^[1]^). There are two topological defects, which form spontaneously during the insertion of the microsphere: a virtual radial hedgehog with topological charge *q*=+1 is formed in the centre of the sphere and a hyperbolic hedgehog defect (blue dot) is formed at a distance $r_{d}$ from the centre of the sphere. The hyperbolic hedgehog has a topological charge of *q*=−1, which balances the topological charge of the virtual radial hedgehog in the centre of the microsphere. (B) Formation of quadrupolar defects (boojums) when a microsphere with tangential surface anchoring is inserted into a nematic liquid crystal (reproduced with permission from ref.^[14]^). These microspheres have two surface boojums located at the interface between the microsphere and the nematic liquid crystal. (C) Formation of a quadrupolar defect (Saturn ring) when a microsphere with homeotropic surface anchoring is inserted into a thin planar aligned cell filled with a nematic liquid crystal (reproduced with permission from ref.^[15]^). Saturn rings may also arise from colloidal particles with weak surface anchoring.

According to Musevic,^[1]^ a small microsphere with homeotropic surface treatment, dispersed in a nematic LC, forces the mesogens to align perpendicularly at all points of the closed surface (as shown in Figure 1A). The LC molecules are naturally aligned homogeneously and the introduction of the microsphere with homeotropic anchoring creates a disturbance in the LC director field. The microsphere acts as a q=+1 topological defect, which cause a q=-1 defect to form in the vicinity (as shown in Figure 1A), to ensure conservation of the overall topological charge. These two charges form a topological dipole which is attributed to the presence of the colloidal microsphere.[^1^, ^13^] In contrast to dipolar colloidal particles, quadrupolar topological defects (boojums and Saturn rings) in nematic LCs are created when microspheres with tangential anchoring are dispersed in a nematic LC or when the surface anchoring on the dispersed microspheres is weak.^[1]^ The tangentially anchored microspheres are expected to interact as quadrupoles with two surface boojums located at the interface between the microsphere and the nematic liquid crystal (see Figure 1B).[^1^, ^14^] The Saturn ring defect (see Figure 1C) around a colloidal particle with homeotropic surface anchoring, dispersed in a planar aligned nematic LC, can be observed if the particle has weak surface anchoring or the planar cell in which the LC is filled is only slightly larger (i. e. the difference should be ≈0.2μm ) than the particle diameter size.[^1^, ^15^] Linear chains of colloids in LCs are formed due to dipolar interactions, while zigzag‐shaped chains are formed due to quadrupolar interactions, as observed with Saturn rings.^[15]^ Defects in nematic LCs need not only be created by inclusion of colloidal particles but can also be observed from isotropic droplets in a nematic environment obtained by heating the LC to the nematic‐isotropic two‐phase region, as shown in the work of Dolganov et al.^[16]^ In the latter case the nematic E7 was filled into a glass cell with homogeneous alignment and heated to create isotropic droplets which nucleate and grow. Nucleation of the droplets was observed on the top and the bottom surfaces of the cell. As the size of the droplets increased and their boundaries contacted both surfaces of the cell, the droplets flattened. When heating was stopped, droplets with static defect configuration were observed. The static structure and dynamics of topological defects were investigated on the interface of the droplets and on the straight phase boundary between the nematic and isotropic phases. The studies also showed that the Saturn ring defect which existed for small droplets was transformed for large droplets into two defects localized in the vicinity of the droplet surface.^[16]^

Dipolar colloidal‐sized inclusions were first reported by Poulin et al.^[17]^ where small water droplets were introduced into the nematic 5CB with the surfactant sodium dodecyl sulphate (SDS). The isolated water droplets spontaneously formed chains which were directed along the far‐field director. The number of induced topological defects by a system of N water droplets was N‐1, further emphasizing the intricate interplay between the LC and colloidal entities. The spontaneous formation of colloidal chains in the nematic LCs is a clear indication of the forces between the colloidal particles. The tensorial nature of the ordering field in the LC mediates the colloidal interactions. If two colloidal particles are brought near each other, their regions of elastic distortion will begin to overlap, and the total free energy of the LC is dependent on the separation of the particles.^[18]^ When the elastic distortion energetically lowers the total free energy of the system, the particles will tend to share that overlapping region as much as possible.[^1^, ^14^] Due to the orientational long‐range order of the nematic LC, the elastic distortion is also of long range and varies as a power law with distance. For quadrupolar particles, the elastic distortion decays with distance from the surface and vanishes at approximately one particle diameter.

The first experimental confirmation of the power law dependence of the force between dipolar colloidal particles in a nematic LC was provided by Poulin et al.^[19]^ In their experiment, ferrofluid droplets were dispersed in the nematic 5CB along with the surfactant polysorbate 60 (Tween‐60). The ferrofluid droplets resembled microparticles dispersed in nematic LCs exhibiting dipolar interactions. They measured the attractive force between two droplets as a function of time, after the external magnetic field was turned off. The attractive forces between colloidal particles were determined by using the droplet velocity as a function of the separation between two droplets. The magnitude of this force was found to be $F_{a}\left(r\right)=\frac{CKa^{4}}{r^{4}}$ , where r is the distance between the centres of the droplets, C is a constant, K is the elastic constant of the LC in single constant approximation and *a* is the droplet radius. For a≈4μm and in the one constant approximation with *K*=10 pN, the value of C was found to be *C*=70. For dipolar particles, while the magnitude of the force between two droplets separated by a hedgehog defect is inversely proportional to the fourth power of the separation, the magnitude of the force between two droplets separated by a so‐called bubble‐gum defect is constant (≈1.5 *K*). The work reported by Poulin et al. was further verified by Dierking et al.^[7]^ when they studied the bubble‐gum defect between two ferrofluid droplets dispersed in 5CB. The elastic interactions between the droplets are balanced by the Stokes drag, while the inertial forces are neglected. When two droplets are separated by a bubble‐gum defect, elastic interactions cause an attractive force between the droplets in the absence of a magnetic field, linearly decreasing their separation with time. The force between two ferrofluid droplets was obtained to be approximately 37 pN (using the viscosity of 40 mPa s, parallel to the director field) which is comparable to the result obtained by Poulin et al.[^7^, ^18^]

The experimental work of Poulin et al.^[19]^ and Dierking et al.^[7]^ highlighted the role of defects in the formation of droplet chains. Lubensky et al.^[20]^ used the Frank free energy approach and theoretically investigated the distortions within the nematic LC by dispersing water droplets coated with a surfactant. Using various variational approaches, they demonstrated that a single droplet created a hedgehog defect out of the nematic host to form a tightly bound dipole in the lowest energy configuration, while configurations in which the droplet was encircled by a disclination ring had higher energy. A phenomenological model in which the topological dipoles were coupled to the nematic director via a flexoelectric interaction was used to derive the effective long‐range dipolar interaction between water droplets which led to chaining.^[20]^ The solvent‐mediated dipolar interactions can elegantly explain the experimentally observed chaining, enhancing the understanding of the long‐range attractive forces at play.

The versatility of dispersing ferrofluid droplets was illustrated further in the work of Fan et al.^[21]^ on micro‐robots composed of ferrofluid droplets dispersed in water and studying the effect of the surfactant sodium dodecyl sulfate (SDS) on the system. These micro‐robots were controllable both individually and collectively with multiple programmable deformations which opens new possibilities in the field of untethered micromanipulation and targeted cargo delivery.

Here, we synthesize ferrofluid droplet chains in nematic LC 5CB and study the dynamic behaviour of the droplet chains when subjected to an external magnetic field within the LC medium. The application of a small magnetic field to the system induces coordinated movement of entire droplet chains without disrupting their structural integrity or causing a director reorientation via the magnetic Freedericksz effect. The study explores the velocity dependence on the chain length (number of droplets) and the anisotropic properties inherent in the LC environment. The study is extended to the investigation of ferrofluid chains coated with the surfactant Tween 60, presenting and analysing their behaviour comparatively to the uncoated ferrofluid chains. The magnetic colloidal particles in the ferrofluid located at the interface between the ferrofluid and the LC arrange so that the boundary layer has a net zero contribution to the magnetization of the ferrofluid droplets.^[7]^ We also estimate the boundary layer thickness for each chain size, for both surfactant coated and uncoated droplet chains. Understanding the boundary layer for ferrofluid droplet chains is crucial for comprehending the broader implications of the ferrofluid‐LC systems.^[22]^

## 2 Materials and Methods

The method for creating uncoated ferrofluid droplets involved mixing the water‐based ferrofluid WHKS1S12 (Liquid Research Ltd, UK) with the thermotropic nematic room temperature liquid crystal 5CB (Fluorochem). The mean elongated length of the solid needle‐shaped magnetic nanoparticles within the ferrofluid was approximately 10 nm.^[23]^ The ferrofluid was added to the LC and the mixture was vortexed using an IKA Vortex Genius 3 mixer to obtain a homogeneous emulsion. This method generated *polydisperse* droplets in the LC, which formed linear chains due to the dipolar interactions. In order to ensure synergetic properties, the concentration of the ferrofluid in a LC matrix was always maintained below 3 % by volume.[^3^, ^22^]

**Figure 2:**
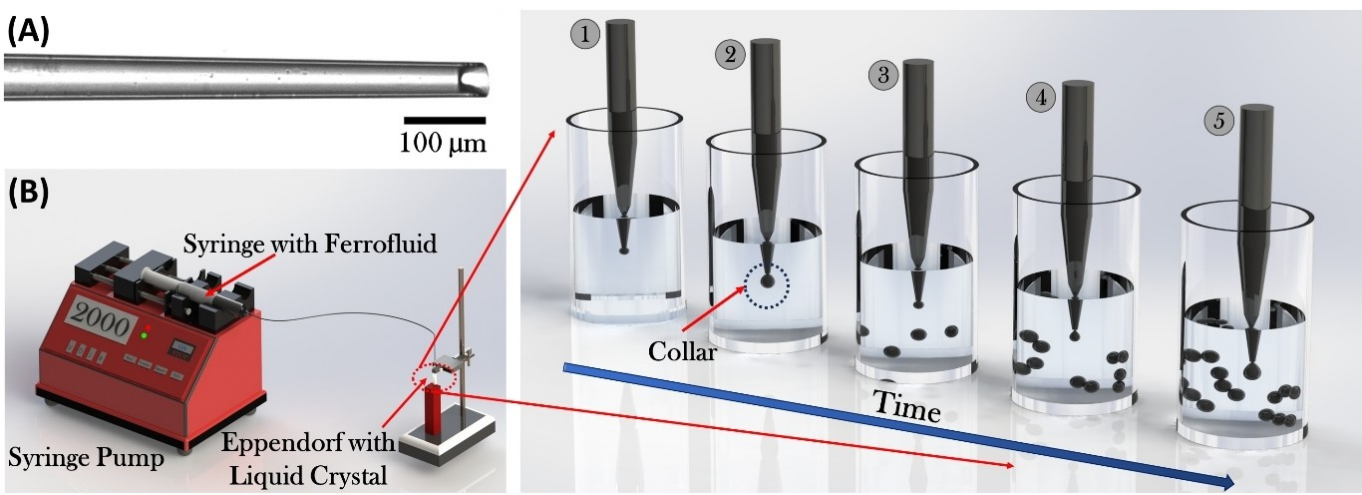
(A) Image of the irregularly broken glass capillary tip. (B) Schematic of micro‐syringe setup. A thin glass capillary tube is filled with the ferrofluid which is ejected with a flow rate of 200 nL/hr into an Eppendorf vial containing a mixture of 5CB with surfactant Tween‐60. The end of the tube is connected to an irregularly broken pipette tip from which monodisperse ferrofluid droplets are produced. Note: The nematic 5CB is not transparent but is represented as so for the visibility of the ferrofluid droplets. The images sequence 1–5 illustrate the process of collar formation leading to the creation of single and chains of droplets within the Eppendorf tube.

To create *monodisperse* ferrofluid droplets, the microfluidic snap‐off approach of Barkley et al.^[24]^ was utilised. A syringe connected to a glass capillary was filled with the ferrofluid and the magnetic fluid was slowly injected into an Eppendorf tube which consists of a mixture of 5CB with approximately 1 % (w/v) surfactant Tween‐60 (prepared by vortexing). We ensured that the tip of the glass capillary was broken‐of irregularly (as depicted in Figure 2A) to utilize the snap‐off mechanism to create monodispersed ferrofluid droplet chains in the LC‐surfactant mixture. The ferrofluid containing syringe was connected to a syringe pump (NE‐4002X SyringeTWO), and the flowrate was maintained constant at 200 nL/hr to ensure quasi‐static conditions. The addition of surfactant to the LC not only produced homeotropic surface anchoring on the droplets, but also reduced the tendency of coalescence of the droplets in the continuous phase. Initially, the LC wets the capillary, resulting in the formation of a thin wetting film which coats the inside walls of the capillary. The ferrofluid is now gradually injected into the LC‐surfactant system through the tip of the glass capillary, resulting in the formation of a droplet. As the droplet grows within the Eppendorf tube, the balance of interfacial forces between the ferrofluid and LC‐surfactant phase leads to the formation of a collar. The collar is defined to be the point at which the radius of the ferrofluid within the tube is minimal. Subsequently, the ferrofluid droplet snaps off from the capillary tube and the process repeats. A schematic illustration of the micro‐syringe setup is depicted in Figure 2B.

**Figure 3:**
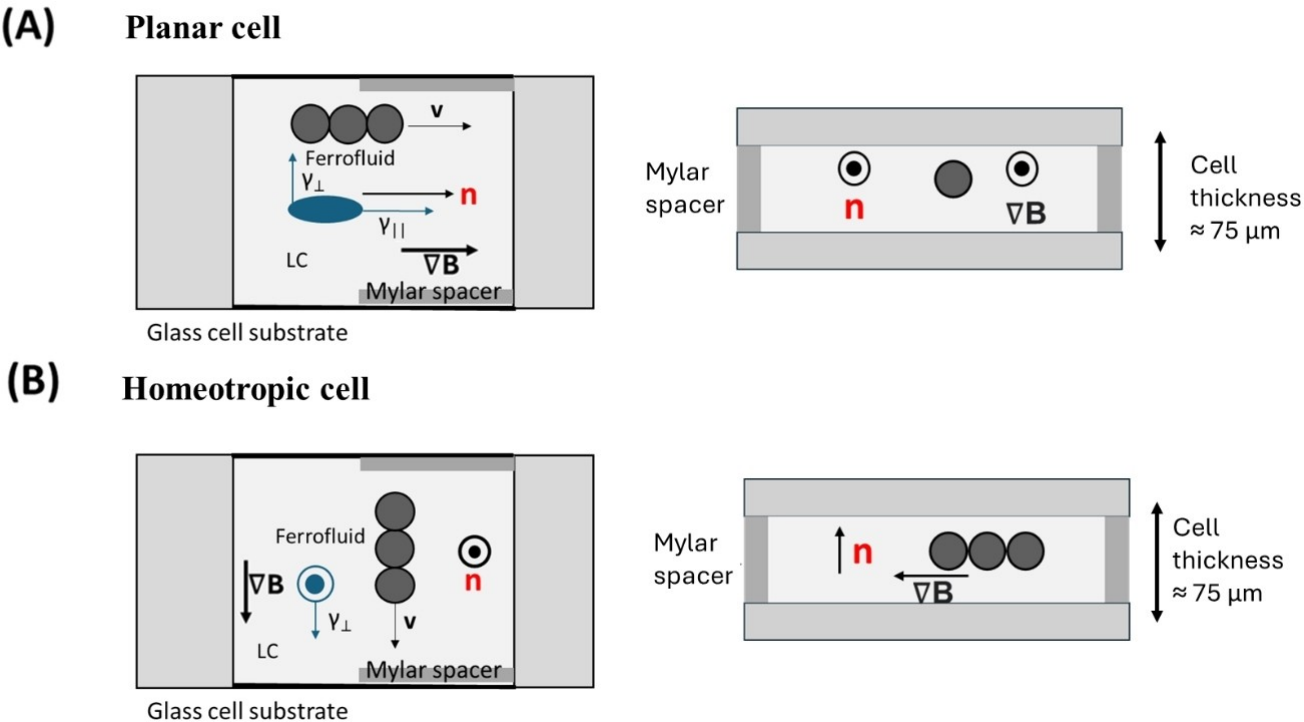
Experimental investigation of ferrofluid droplet chains in (A) planar and (B) homeotropic cells; top view and side view respectively. For planar aligned cells, measurements can be conducted both parallelly and perpendicularly to the director while for homeotropically aligned cell, measurements can only be conducted perpendicularly to the director. Here, **n** is the director, **v** the chain velocity, and γ_||_ and γ_⊥_ the viscosities parallel and perpendicular to the director of the LC.

The droplet chains that were created using both the above‐mentioned processes were now used to investigate their magnetophoretic response within a LC ambiance. Therefore, glass sandwich cells are prepared with substrates coated with polyvinyl alcohol (PVA) or Cetyltrimethylammonium bromide (CTAB) and baked for 20 mins to obtain planar or homeotropic anchoring boundary conditions, respectively (as illustrated in Figure 3). In the case of planar anchoring, the parallel and perpendicular directions are defined with respect to the rubbing direction of the PVA layer. This step was omitted for homeotropic anchoring with CTAB.^[22]^ In CTAB coated cells, measurements can only be done in the direction perpendicular to the director since every direction in the plane of the cell is perpendicular to the director. The reason for using PVA is to obtain a planar aligned LC configuration for measurements to be conducted in directions both parallel and perpendicular to the director. The reason for using CTAB to obtain a homeotropic LC configuration is that the chaining is observed to be more favourable in the homeotropic configuration.

**Figure 4:**
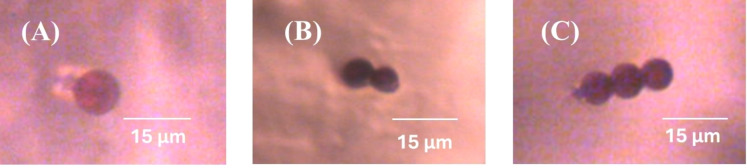
POM image of a (A) single ferrofluid droplet (B) ferrofluid droplet pair (C) three droplet chain in nematic 5CB in glass cells of thickness 75 μm.

The LC‐ferrofluid emulsions prepared by the two methods are filled into sandwich cells of thickness 75 μm by use of a micropipette. The cells are then investigated with a Leica DMLP polarized optical microscope (POM) with a neodymium magnet placed at a fixed distance from the objective to apply an external magnetic field (see Figure 4). The motion of the ferrofluid droplet chains is recorded using an IDS camera (UI‐3360CP−C‐HQ, uEye Gigabit Ethernet) and the positions are measured using the Trackmate plugin in ImageJ‐FIJI v1.54 (frame rate=10 fps, image resolution=2048×1048). When the magnetic field is applied, the initial transients such as the reorientation and acceleration of the droplets are neglected, and measurements are taken when the uniform terminal velocity is reached by the droplets. From the linear dependence of position as a function of time, the terminal velocity is determined in directions both parallel and perpendicular to the director field.

**Figure 5:**
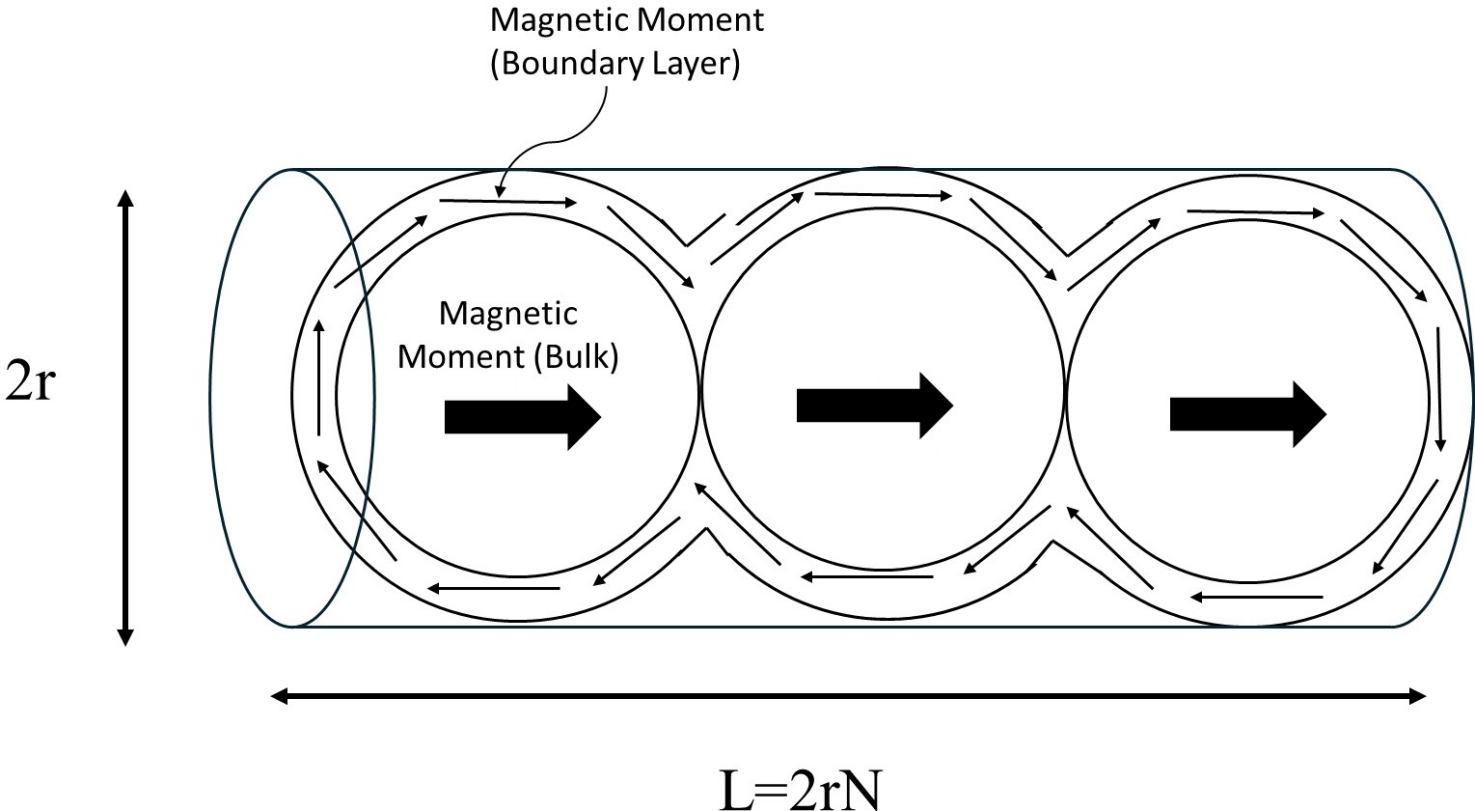
Schematic diagram depicting the ferrofluid droplet chain boundary layer. The boundary layer develops at the LC‐ferrofluid interface so that the magnetic nanoparticles orient either parallel or perpendicular to the interface so that in the boundary layer the net magnetic moment per unit volume compensates to roughly zero. The droplet chain is approximated as a cylinder with length L (L=2rN with N being the number of droplets in the chain and r being the average radius). Shown is the parallel scenario, similarly, this compensation is also observed for perpendicular orientation of the nanoparticles with respect to the interface. (Note: for reasons of clarity, the LC is not shown).

A boundary layer develops at the LC‐ferrofluid interface so that the magnetic nanoparticles orient either parallel or perpendicular to the interface. This orients the magnetic dipole moments so that in the boundary layer the net magnetisation (magnetic moment per unit volume) is roughly equal to zero, while in the bulk it is not (see Figure 5). This description is only a first rough approximation, as it disregards the detailed magnetic dipole moment vector field, which in three‐dimensional space inevitably will exhibit vortices and poles. Nevertheless, this description is valid in first approximation because the nanoparticles carrying the magnetic dipole moments are much smaller (~10 nm) than the scale of the droplets (~10 μm). In the previous work,[^7^, ^22^] the boundary layer for a single ferrofluid droplet dispersed in 5CB was determined by comparing the velocities of different droplets. The boundary layer thickness b was calculated to be approximately b≈4-5μm and only droplets of diameter greater than twice the boundary layer 2b≈9μm , exhibiting motion with the application of a magnetic field. The boundary layer for ferrofluid droplet chains is obtained by approximating the chain as a cylinder and using the resistance coefficient along with the terminal velocity in the drag force equation in the Stokes’ regime. In the absence of surfactants like Tween‐ 60, longer chains of ferrofluid droplets tend to coalesce forming single droplets upon the application of a magnetic field. Tween‐60 is known for its ability to stabilize interfaces and prevent coalescence by forming a protective layer around the droplets thus lowering intermolecular forces and reducing surface tension.[^25^, ^26^]

## 3 Results and Discussion

The uncoated and surfactant coated ferrofluid droplet chain velocities, in the parallel and perpendicular direction with respect to the director, were determined at room temperature. The diameters for the droplets considered are in the range of 8–20 μm in a cell of thickness 75 μm. The uncoated droplets used in our measurement were obtained by vortexing while the coated droplets were obtained using the snap‐off approach. One of the important limitations to the measurements is the more frequent coalescence of the uncoated droplets, which results in obtaining only a few uncoated droplet chains of length 4 or more droplets in both planar cells and in homeotropic cells.

The magnetic force experienced by the droplet is balanced by the Stokes’ drag force as shown in the previous work[^7^, ^22^] and the terminal velocity of single ferrofluid droplets in LCs can be described using Equation (1). The Stokes’ velocity ($v_{eq}$ ) of a single ferrofluid droplet (with volume magnetization $M_{vol}=9450\;Am^{-1}$ as stated by the supplier) of effective radius $r_{eff}$ (radius minus boundary layer thickness) moving through a medium of viscosity γ under the application of magnetic field gradient **∇B** is given by[^7^, ^22^]:

(1) $$v_{eq}=\;\frac{2r_{eff}^{2}M_{vol}.\nabla B}{9\gamma }$$

**Figure 6:**
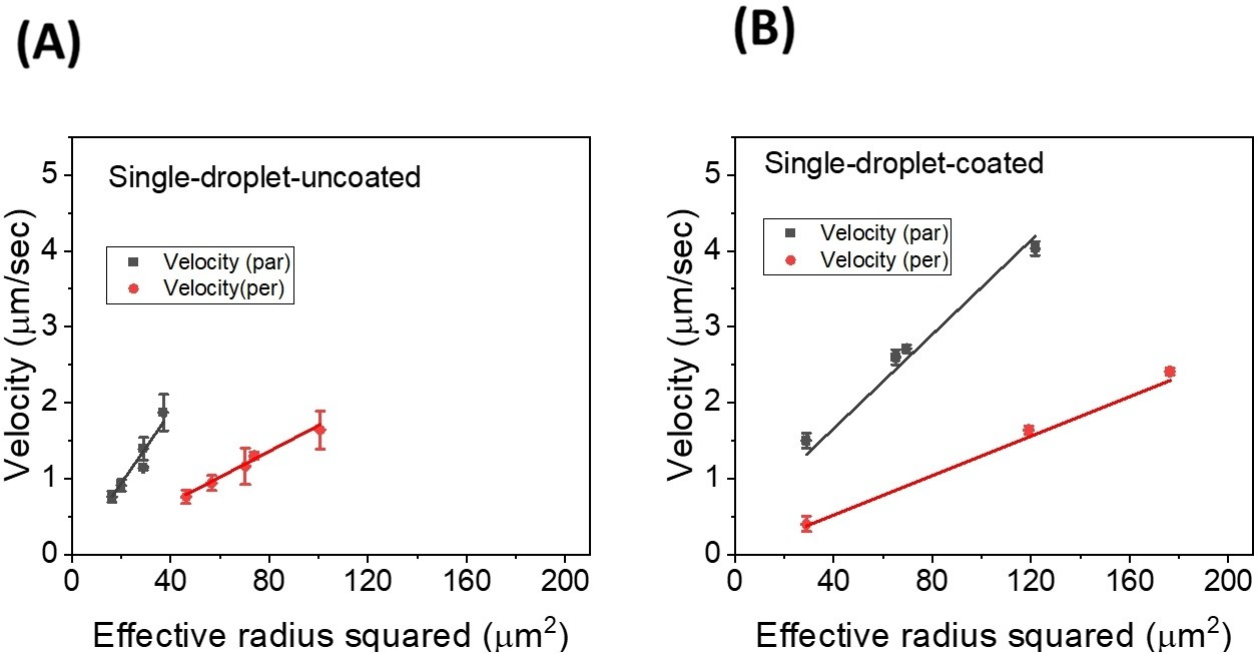
Velocity vs effective radius squared (times magnetic field gradient) for (A) uncoated single droplets and (B) coated single droplets. The solid lines represent the calculated velocity values according to Equation (1) while the symbols represent the measured velocity values. Values for uncoated and coated droplets are very similar in magnitude, while uncoated droplets appear to move slightly faster than their coated counterparts, although the behaviour is close to the limits of error resolution.

Figure 6 shows the experimentally measured velocity plotted against the effective radius square for coated and uncoated single droplets in the parallel and perpendicular direction, along with the calculated velocity using Equation (1). The calculated velocities for uncoated droplets are obtained using the viscosities for 5CB (γ_||_=45±1 mPa s and γ_⊥_=129±4 mPa s) and the boundary layer thickness (5±1 μm) which were experimentally obtained from our previous work^[22]^ along with a measured magnetic field gradient (calibration done using a Hirst GM08 Gaussmeter Hall‐Probe). For coated droplets, the viscosity and the boundary layer thicknesses were measured for single droplets using the method outlined in previous work.^[22]^ The anisotropic viscosity values calculated for 5CB with approximately 1 % (w/v) Tween‐60 are γ_||_=57±5 mPa s and γ_⊥_=147±4 mPa s and the boundary layer value estimates in the range of (6±1 μm). Coating the ferrofluid droplets with an amphiphilic compound thus slightly increases the observed effective viscosities, yet by only about 10 %. The lines in the graph represent the calculated velocities for the effective radius (subtracted with the obtained boundary layer value) determined and the points represent the measured velocity values. While the velocities trend wise exhibit somewhat smaller velocities for the Tween‐60 coated droplets as compared to the uncoated ones, it should be noted that this difference is close to the limit of experimental error and should rather be discussed qualitatively than quantitatively. The behaviour can be explained by the structure of Tween‐60, which consists of a hydrophilic head group that adsorbs on the surface of the aqueous ferrofluid droplet, and a flexible hydrophobic tail which is directed towards the LC side. The incorporation of Tween‐60 thus increases the effective droplet diameter very slightly, but less than could be noticed by molecular length when compared to the droplet radius. Nevertheless, this alteration can affect the effective viscosity of the surrounding LC in the very vicinity of the droplet, as observed from our viscosity measurements for the LC‐surfactant mixture. This increase in viscosity, slows down the motion of the droplets, due to an effectively larger boundary layer. It should be noted though, that we cannot distinguish between the effect of the increased effective viscosity and the thicker boundary layer, because both should slow down the motion of the droplets at otherwise equal conditions.

In our experiments with ferrofluid droplet chains, we observed that the droplet chains collectively move as single objects without breaking and a terminal velocity can be measured for each chain. The drag force on the chain in the Stokes regime follows a linear relationship with the terminal velocity along with a drag coefficient which is related to the size, shape, and orientation of the object. This drag force is balanced by the magnetic force on the chain.^[27]^ The droplet chains can be approximated as cylinders with the length equal to the sum of the droplet diameters in the chain and their diameter being the average diameter of all individual droplets in the chain[^28^, ^29^]:

(2) $$F_{magnetic}=F_{cylindrical\;drag}$$

(3) $${V_{tot}M}_{vol}\nabla B=\frac{4\pi \gamma v_{eq}L_{eff}}{\ln\left(\frac{L_{eff}}{R_{eff}}\right)-0.72}$$

Where V_tot_ is the total volume of the chain, $L_{eff}$ is the effective length of the N droplets in the chain (sum of diameters minus twice the boundary layer) and r_eff_ is the average effective radius (average radius of chain minus boundary layer). From this, the terminal velocity of a chain of N droplets can be calculated as:

(4) $$v_{eq}=\frac{\left(2Nr_{eff}^{3}\nabla B\;M_{vol}\right)\left(\ln\left(\frac{L_{eff}}{r_{eff}}\right)-0.72\right)}{3\gamma L_{eff}}$$

**Figure 7:**
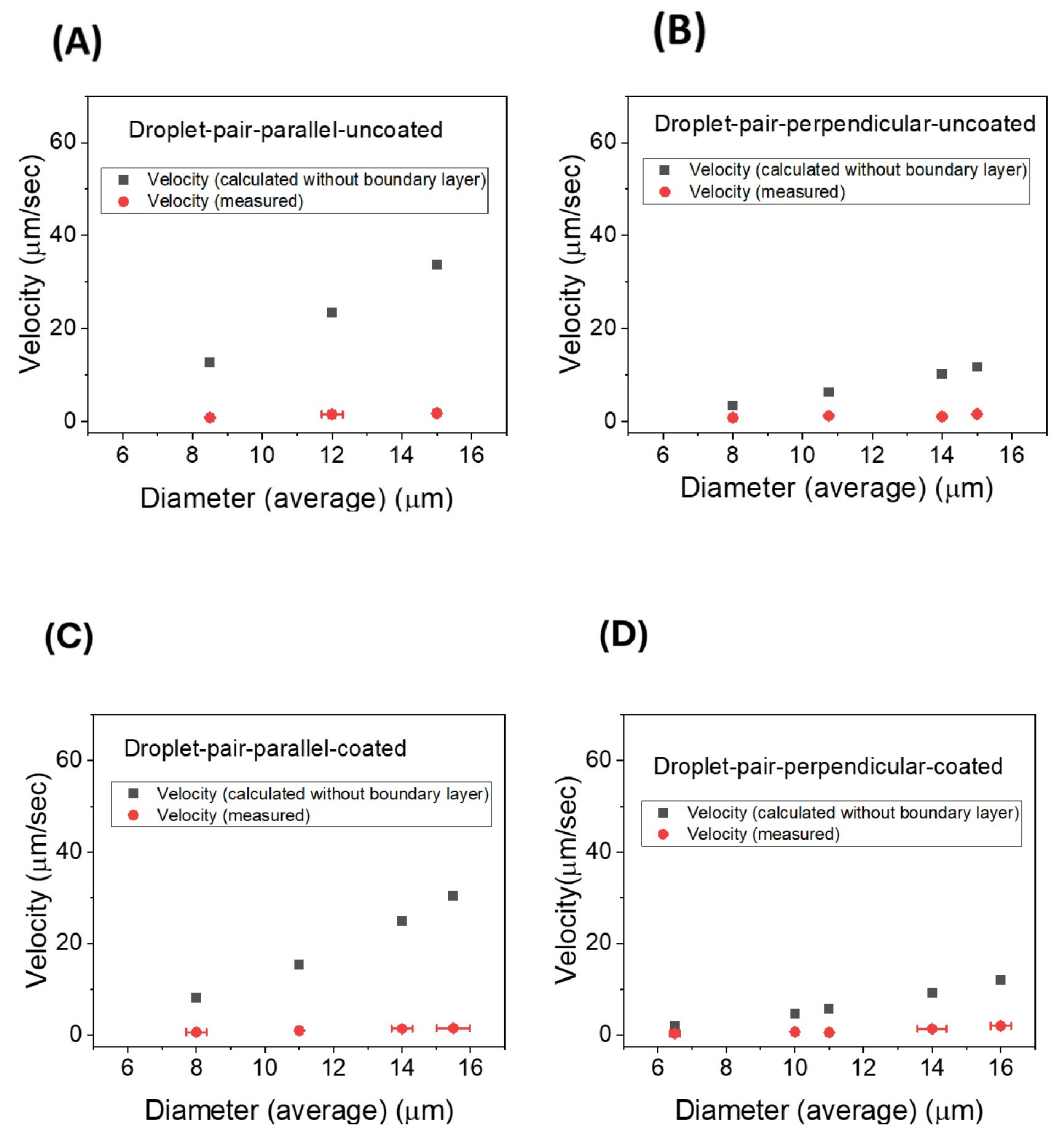
Comparative study of calculated velocity values (without boundary layer) with measured velocity values (∇B=12.835 T/m) for (A) uncoated droplet pairs in the direction parallel to the director, (B) uncoated droplet pairs in the direction perpendicular to the director, (C) coated droplet pairs in the direction parallel to the director and (D) coated droplet pairs in the direction perpendicular to the director.

**Figure 8:**
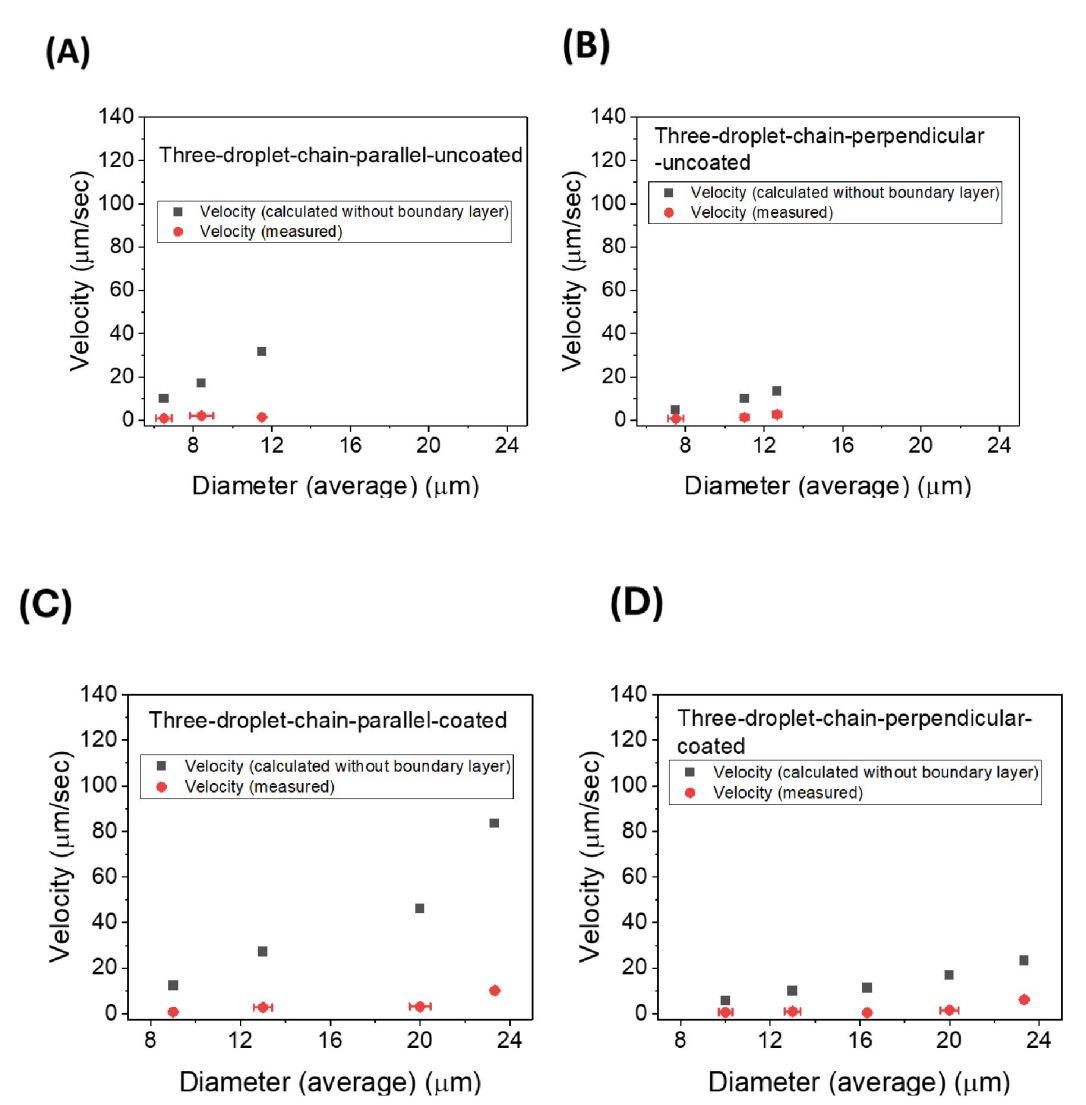
Comparative study of calculated velocity values (without boundary layer) with measured velocity values (∇B≈12 T/m) for (A) uncoated three droplet chains in the direction parallel to the director, (B) uncoated three droplet chains in the direction perpendicular to the director, (C) coated three droplet chains in the direction parallel to the director and (D) coated three droplet chains in the direction perpendicular to the director.

**Figure 9:**
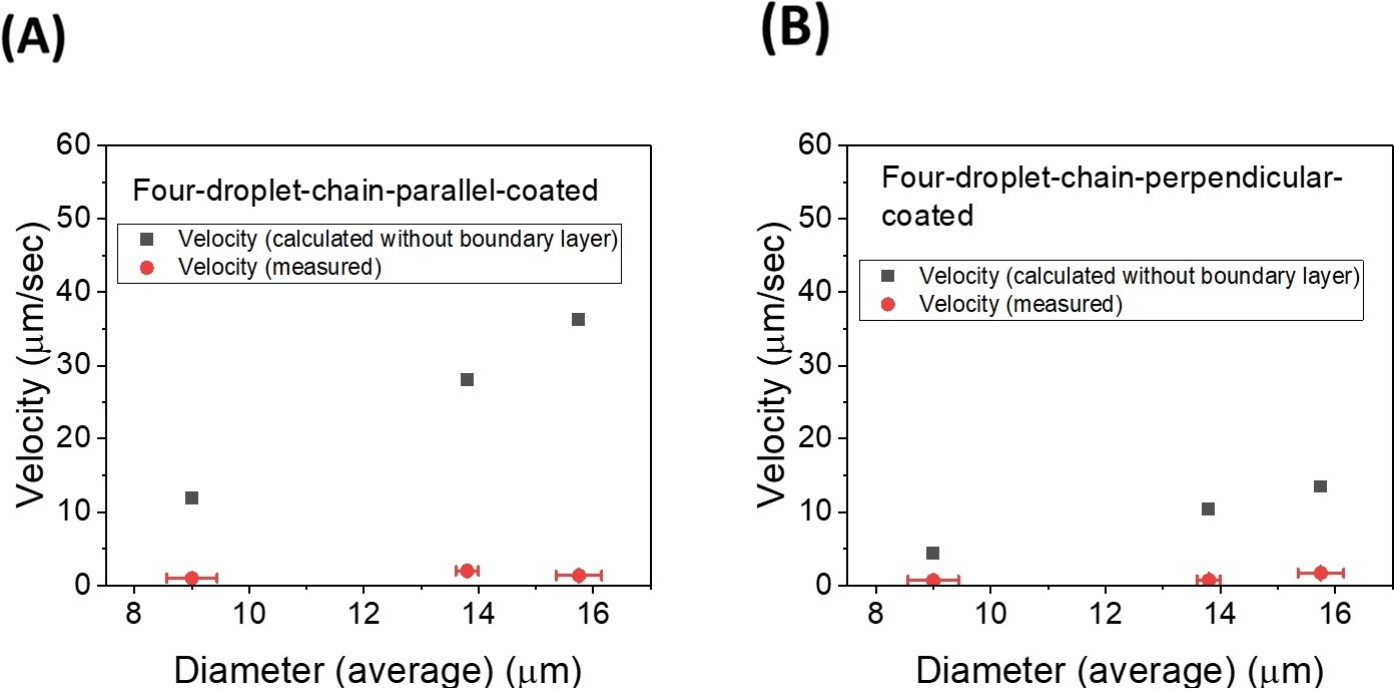
Comparative study of calculated velocity values (without boundary layer) with measured velocity values (∇B=7.239 T/m) for (A) coated four droplet chains in the direction parallel to the director and (B) coated four droplet chains in the direction perpendicular to the director.

The experimentally measured velocity values of droplet chains and the velocity values calculated using Equation (4) (without the boundary layer) are plotted against the respective average diameters of each chain. In Figure 7 the comparison is done for coated and uncoated droplet pairs, in Figure 8 for three‐ and in Figure 9 for four‐droplet chains. Errors indicate the deviations of the droplet diameters from their average and the error in determining the velocities from the linear part of the chain motion. If no error bars are visible, then the errors are smaller than the data point symbol. Data in each graph of a respective figure is plotted on equal scales for easier comparison. Using the viscosity values and the measured magnetic field gradient, the velocity values are calculated, and it is observed that the calculated velocity values are greater in magnitude than the experimentally measured values. This difference can be resolved with the introduction of the boundary layer for chains.

**Figure 10:**
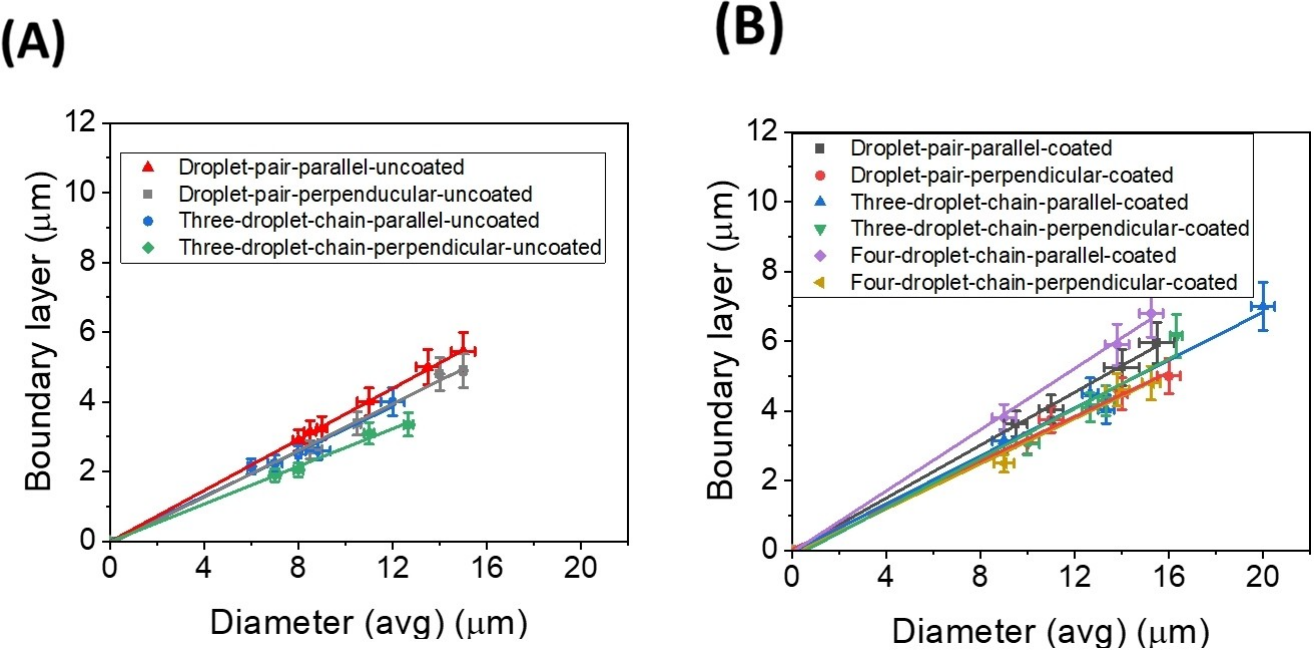
Boundary layer vs Average diameter for (A) Uncoated droplet pairs and three droplet chains in the direction parallel and perpendicular to the director and (B) Coated droplet pairs, three droplet chains and four droplet chains in the direction parallel and perpendicular to the director. A linear relation is observed between the boundary layer thickness and the average diameter.

Figure 10 shows the variation of boundary layer values with average diameter for both coated and uncoated droplets. Chains of average droplet diameters below approximately 7 μm for both the uncoated and coated droplets do not exhibit motion on application of the magnetic field, indicating that the estimated values are reasonable, because the boundary layers do not contribute to the bulk magnetisation of the droplets or droplet chains. The boundary layer shows a linear relation with the average diameter for droplet pairs and three droplet chains for both the coated and uncoated cases. Within the limits of error the determined boundary layer thickness for parallel and perpendicular motion with respect to the director, are equal. Seemingly, longer chains have slightly thinner boundary layers. Yet, we again have to note that these observations are more of a qualitative than quantitative nature, because much of the effects are very close to the limit of error resolution.

**Figure 11:**
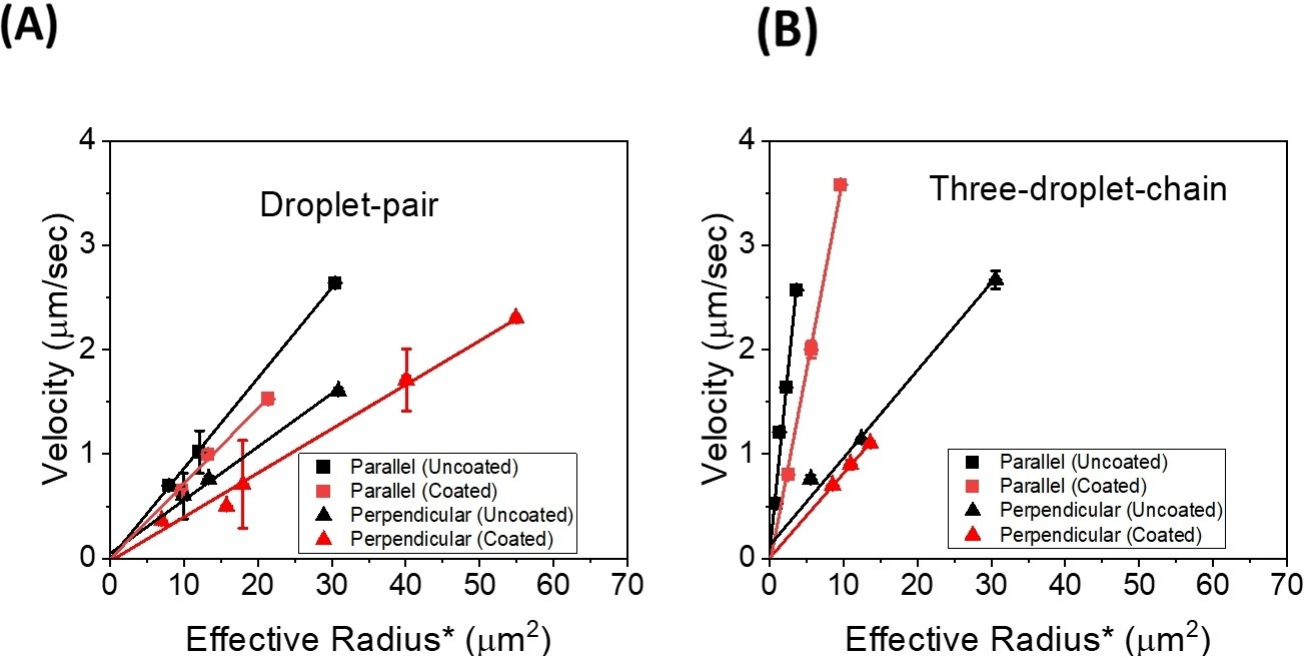
Velocity vs effective radius* (RHS of Equation (4) for specific magnetic field gradient, viscosity values and M_vol_=9450 A/m) (A) uncoated droplet pairs and droplet pairs coated with Tween‐60 in parallel and perpendicular direction and (B) uncoated three droplet chains and three droplet chains coated with Tween‐60 in parallel and perpendicular direction. (∇B≈12 T/m).

Figure 11(A) shows a comparative representation of the velocities of droplet pairs for both coated and uncoated cases plotted against the respective combined effect of effective radius, chain size and resistance factor as specified in Equation (4). The comparative study shows that the coated droplet pairs move with a lower velocity than the uncoated droplet pairs due to the increased viscosity and boundary layer thickness. The same behaviour can be observed for three droplet chains as seen in Figure 11(B).

Figure 11 indicates that not only the velocities parallel to the director are larger than the ones perpendicular, but also that uncoated droplet chains are moving faster than Tween‐60 coated ones. This can be explained by the slightly enlarged boundary layer for the coated chains, and the fact that the flexible ends of the Tween‐60 molecules, sticking into the liquid crystal, will increase the effective viscosity in the vicinity of the chains, thus leading to slower velocities. Overall, longer droplet chains exhibit a larger velocity, because of the increased bulk magnetisation.

## 4 Conclusions

The velocities of ferrofluid droplet chains in a nematic LC were measured in both parallel and perpendicular directions to the director. The ferrofluid droplets behave, in first approximation, like microparticles, causing dipolar defects in the director field. The attraction between these defects leads to chaining of ferrofluid droplets. With the application of an external magnetic field (below the Freedericksz threshold) to the LC‐ferrofluid system, the entire ferrofluid droplet chain moves without being destroyed, indicating the structural integrity of the system.

Single ferrofluid droplets in LCs follow Stokes’ law considering that the droplet is spherical. The droplet chains are approximated as cylinders with the length equal to the sum of the droplet diameters in the chain and the diameter being equal to the average diameter of all the droplets in the chain. The viscous drag force can then be determined from the terminal velocity and the magnetic field gradient using the formula for resistance of cylinders in the Stokes’ regime.[^28^, ^29^] A comparison of experimentally measured velocity values with those calculated theoretically, at different magnetic field gradients, is presented. The calculated velocity values are larger in magnitude than the measured ones. This difference can be resolved with the introduction of a boundary layer at the LC‐ferrofluid interface where the magnetic nanoparticles in the boundary layer are oriented such that the net magnetic moment is roughly zero in first approximation when averaging over the entire boundary layer. The boundary layer thickness appears to vary linearly with the average diameter for both coated and uncoated droplet pairs and chains. It is observed that the average boundary layer thickness of the surfactant coated chains is slightly larger than that of the uncoated chains which can be attributed to the adsorption of surfactant molecules at the ferrofluid‐LC interface.

It was further observed that droplets coated with amphiphilic Tween‐60 move slower than their uncoated equivalent. This can be explained by the increase of the boundary layer thickness and the effective LC viscosity in the vicinity of the flexible hydrophobic chains being directed into the liquid crystal leading to distortions in the local director field. Droplets can be stabilized, and coalescence is largely reduced by the inclusion of surfactants such as Tween‐60. This study provides novel insights into the stability and functionality of ferrofluid‐doped LCs and could pave the path towards development of novel magnetically functionalised LC systems for sensing applications.

## Conflict of Interests

The authors declare no conflict of interest.

5

## Data Availability

The data that support the findings of this study are available from the corresponding author upon reasonable request.
